# StarD5 levels of expression correlate with onset and progression of steatosis and liver fibrosis

**DOI:** 10.1152/ajpgi.00024.2024

**Published:** 2024-04-09

**Authors:** Genta Kakiyama, Kei Minoiwa, Nanah Bai-Kamara, Taishi Hashiguchi, William M. Pandak, Daniel Rodriguez-Agudo

**Affiliations:** ^1^Department of Internal Medicine, Virginia Commonwealth University School of Medicine, Richmond, Virginia, United States; ^2^Research Services, Central Virginia Veterans Affairs Healthcare System, Richmond, Virginia, United States; ^3^Department of Pediatrics, Juntendo University Faculty of Medicine, Tokyo, Japan; ^4^Research and Development Bureau, SMC Laboratories, Inc., Tokyo, Japan

**Keywords:** cholesterol, fatty liver, insulin resistance, transporter, triglycerides

## Abstract

Insufficient expression of steroidogenic acute regulatory-related lipid transfer protein 5 (StarD5) on liver cholesterol/lipid homeostasis is not clearly defined. The ablation of StarD5 was analyzed in mice on a normal or Western diet (WD) to determine its importance in hepatic lipid accumulation and fibrosis compared with wild-type (WT) mice. Rescue experiments in *StarD5*^−/−^ mice and hepatocytes were performed. In addition to increased hepatic triglyceride (TG)-cholesterol levels, global *StarD5*^−/−^ mice fed a normal diet displayed reduced plasma triglycerides and liver very low-density lipoprotein (VLDL) secretion as compared with WT counterparts. Insulin levels and homeostatic model assessment for insulin resistance (HOMA-IR) scoring were elevated, demonstrating developing insulin resistance (IR). WD-fed *StarD5*^−/−^ mice upregulated WW domain containing transcription regulator 1 (TAZ or WWTR1) expression with accelerated liver fibrosis when compared with WD-fed WT mice. Suppression of oxysterol 7α-hydroxylase (CYP7B1) coupled with chronic accumulation of toxic oxysterol levels correlated with presentation of fibrosis. “Hepatocyte-selective” StarD5 overexpression in *StarD5*^−/−^ mice restored expression, reduced hepatic triglycerides, and improved HOMA-IR. Observations in two additional mouse and one human metabolic dysfunction-associated steatotic liver disease (MASLD) model were supportive. The downregulation of StarD5 with hepatic lipid excess is a previously unappreciated physiological function appearing to promote lipid storage for future needs. Conversely, lingering downregulation of StarD5 with prolonged lipid-cholesterol excess accelerates fatty liver’s transition to fibrosis; mediated via dysregulation in the oxysterol signaling pathway.

**NEW & NOTEWORTHY** We have found that deletion of the cholesterol transport protein StarD5 in mice leads to an increase in insulin resistance and lipid accumulation due to the upregulation of lipid synthesis and decrease VLDL secretion from the liver. In addition, deletion of StarD5 increased fibrosis when mice were fed a Western diet. This represents a novel pathway of fibrosis development in the liver.

## Graphical abstract

**Figure:**
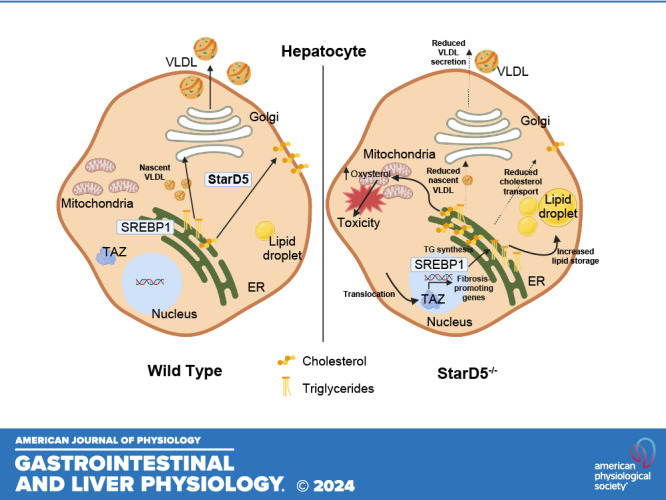

## INTRODUCTION

Cholesterol excess has long been hypothesized as a mediator of inflammation and fibrosis within the liver. However, clear mechanistic pathways to explain the role of cholesterol or its metabolites have remained unclear. Recently, a direct role of excess endoplasmic reticulum (ER) cholesterol in the transcriptional activation of fibrotic genes was described (1). More recently, it was shown in the setting of insulin resistance (IR) that accumulation of toxic levels of cholesterol metabolites is responsible for “initially” driving inflammation (2), with subsequent inflammatory cell infiltrate (3) and cascading events. This paper outlines an intracellular cholesterol binding protein’s novel physiological function and explores its regulation in mediating hepatocellular toxicity and ensuing fibrosis in metabolic dysfunction-associated steatotic liver disease (MASLD).

Cholesterol is an essential molecule for the correct structure and functionality of all cellular membranes. Its distribution between membranes varies greatly, creating a gradient of cholesterol concentration from the ER (with the lowest amount of cholesterol) to the plasma membrane (PM) (with the highest amount of cholesterol) with varying levels of cholesterol among other organelle membranes (4). The ER is the cell’s main organelle for cholesterol synthesis and redirection, and its transport out of the ER becomes essential in maintaining the cell’s integrity as well as its cholesterol homeostasis. More selectively, sterol (cholesterol and metabolites) levels have to be maintained within a narrow range in the ER, being achieved by a feedback mechanism that revolves around the transcription factor sterol regulatory-element binding protein 2 (SREBP-2) and the sterol-sensing protein SREBP cleavage-activating protein (SCAP) (5). The disruption of the lipid homeostasis in the ER can trigger ER stress and subsequently activation of the unfolded protein response (UPR) (6–8), leading to *de novo* lipogenesis (9), reduced fatty acid oxidation, disturbed lipoprotein and very low-density lipoprotein (VLDL) secretion (10), and increases in VLDL receptor (VLDLR) expression (11) resulting in steatosis. In the specific case of cholesterol, its *de novo* synthesis during ER stress comes from the activation of SREBP-2 (12). When disruption in cholesterol metabolism leads to an accumulation of free cholesterol within the ER, it either directly or indirectly drives the progression of fatty liver to inflammation through upregulation of TAZ (1), a transcriptional regulator that promotes fibrosis. Therefore, the regulation of the levels of cholesterol is extremely important for the homeostasis of organelles, cells, and tissues.

Cholesterol, along with lipids, is exported from the ER as part of secretory vesicles or by lipid transfer proteins (LTPs) (4). One family of LTPs, the steroidogenic acute regulatory-related protein (StAR)-related lipid transfer family (13, 14), is key to their movement between membranes. All the proteins in the family contain a conserved steroidogenic acute regulatory protein-related lipid transfer (START) domain, characterized by a hydrophobic sterol-binding pocket (15). StAR/steroidogenic acute regulatory-related lipid transfer domain 1 (StarD1) is the archetype of the family and includes a mitochondrial targeting sequence, being responsible for the transfer of cholesterol to the mitochondria (16). Other members include the phosphatidylcholine transfer protein (PCTP/StarD2) (17) and StarD3, which has a transmembrane domain that enables the protein to transfer cholesterol between the ER and endosomes (18). A unit of the StAR-related lipid transfer family is the StarD4 subfamily, composed of the StarD4, StarD5, and StarD6 proteins (14). The proteins in this subfamily are characterized for only having the START domain of around 220 amino acids (13). Of these proteins, StarD5 has been shown to be regulated by ER stress and able to bind cholesterol and 25-hydroxycholesterol (25-HC) (19, 20). It is widely expressed at high levels in immune-related cells (21), but onset of ER stress and activation of the UPR pathway leads to increased expression in all cells, including hepatocytes (20, 22). Recent studies from our laboratory, using a StarD5 knockout (*StarD5*^−/−^) mouse, have shown that ablation of StarD5 protein leads to a decrease in PM cholesterol and cholesterol efflux in macrophages (22). This is accompanied by an increase in intracellular cholesterol and “unexpected” dramatic increase in neutral lipid accumulation. Isolated hepatocytes displayed an increase in PM-accessible cholesterol as well under ER stress conditions. Conversely, as compared with controls, *StarD5*^−/−^ cells demonstrated lower levels of PM cholesterol with an expected increase in PM fluidity determined using phasor-fluorescence lifetime imaging microscopy (FLIM) of LAURDAN fluorescence analysis (22).

Based on the results presented here, the ablation of StarD5 uncovered an unexpected effect on lipid metabolism in the liver. In addition to its known ability to provide a protective response with cell ER stress, StarD5 appears to play a housekeeping function in maintaining cholesterol and triglyceride (TG) homeostasis. Chow-fed mice lacking StarD5 expression displayed not only liver triglyceride accumulation but also IR, decreased VLDL secretion, and dysregulation in genes of the fatty acid synthesis pathway explaining the accumulation of lipids in the liver. Restoring StarD5 expression in the liver of *StarD5*^−/−^ mice improved IR and reduced liver triglycerides. StarD5’s downregulation in response to abundant lipid supply led to physiologic-designed triglyceride storage, but with more chronic overabundance, as with Western diet (WD) feeding, led to the accumulation of toxic cholesterol metabolites and the acceleration of liver fibrosis. These results indicate the important role of StarD5 in maintaining the lipid homeostasis of the liver, which has implications for IR and dyslipidemia in the liver, such as MASLD and metabolic-associated steatohepatitis (MASH).

## MATERIALS AND METHODS

### Materials

The Infinity Triglycerides assay kit, the NE-PER Nuclear and Cytoplasmic Extraction Reagents kit, and SuperSignal West Pico Chemiluminescent Substrate were from Thermo Fisher Scientific. Antibodies used for immunoblots are listed in Table 1. RNeasy Plus Mini Kit was from Qiagen. Ad-CMV-hStarD5.MycDDK was prepared by Vector Biolabs using a plasmid (from OriGene) encoding the human StarD5 cDNA fused to myc and flag tags driven by the cytomegalovirus (CMV) promoter. Adeno-associated virus AAV9-TBG-hStarD5.MycDDK was also prepared by Vector Biolabs using the same plasmid described earlier but driven by the thyroxine binding globulin (TBG) promoter (for liver specific expression). Control Ad-CMV-enhanced green fluorescent protein (eGFP) and AAV9-TBG-eGFP were also from Vector Biolabs. *d*_6_-25-HC (26,26,26,27,27,27-[^2^H_6_]-25-HC) was purchased from Cayman Chemical (Ann Arbor, MI), and 25,26,26,26,27,27-[^2^H_6_]-26-HC (*d6*-26-HC) and 23,23,24,24,24-[^2^H_6_]-3β-hydroxy-5-cholesten-(25R)26-oic acid (*d5*-3βHCA) were available from Avanti Polar Lipids (Alabaster, AL).

**Table 1.** Antibodies used in this study

Antibody	Company Name (Reference)	Dilution
Anti-FLAG	Sigma (F7425)	1:4,000
Anti-ACC	Abcam (ab45174)	1:1,000
Anti-FAS	Abcam (ab22759)	1:1,000
Anti-StarD5	Santa Cruz (sc-514236)	1:400
Anti-CYP7B1	Abcam (ab138497)	1:1,000
Anti-GAPDH	Cell Signaling (2118S)	1:2,000
Anti-TAZ	Abcam (ab110239)	1:1,000
Anti-Apolipoprotein B	Abcam (ab20737)	1:500
Anti-Calnexin	Millipore/Sigma (MAB3126)	1:1,000
Anti-SREBP1	Thermo Fisher Scientific (MA5-11685)	1:1,000
Anti-Lamin B1	Abcam (ab16048)	1:1,000
Goat anti-rabbit HRP-conjugated IgG	Bio-Rad (170-6515)	1:2,000
Goat anti-mouse HRP-conjugated IgG	Bio-Rad (170-6516)	1:2,000

ACC, acetyl CoA-carboxylase; CYP7B1, oxysterol 7α-hydroxylase; FAS, fatty acids synthetase; HRP, horseradish peroxidase; SREBP1, sterol regulatory-element binding protein 1.

### Animal Studies

#### StarD5^−/−^ mice model.

Animal studies and care were performed under the guidelines of the Virginia Commonwealth University and Central Virginia Veterans Affairs Healthcare System (Richmond VA Medical Center) Institutional Animal Care and Use Committees (IACUC), in accordance with the principles and procedures outlined in the National Research Council *Guide for the Care and Use of Laboratory Animals* under Assurance Number A3281-01. Animals were housed in individually ventilated cages in a barrier vivarium, which excludes all known mouse viruses and parasites and most bacteria including helicobacter.

*StarD5*^−/−^ mice were generated on C57BL/6 background using the CRISPR/Cas9 approach as described previously (22). *StarD5*^−/−^ mice (8-wk-old male) were fed a standard mouse chow [normal diet (ND), Teklad LM-485] or a Western diet (WD: Teklad TD.88137) containing 0.2% (wt/wt) cholesterol and 21% total fat (42% kcal from fat, >60% from saturated fatty acids, and 34% from sucrose) for 18 wk ad libitum. For controls, age, sex, and weight matched C57BL/6J [wild-type (WT)] mice fed ad libitum with ND and WD were used.

For the gain-of-function study, AAV9-TBG-hStarD5.MycDDK and control AAV9-TBG-eGFP were administered to *StarD5*^−/−^ mice or C57BL/6J (8-wk-old males) by retro-orbital vein injection (1 × 10^11^ genome copies/mouse). They were fed ad libitum ND and autoclaved water for 3 wk.

All mice were fasted for 4 h before they were euthanized. Mice were euthanized under isoflurane anesthesia for collection of liver tissue. A portion of the liver was fixed in 10% neutral-buffered formalin for histology analysis. The remaining portion was snap frozen in liquid nitrogen and stored at −80°C until further analysis. Blood was also simultaneously collected, and serum was collected by centrifugation at 1,620 *g* for 15 min at 4°C.

#### Streptozotocin–high-fat diet-induced MASH-fibrosis mice model.

The streptozotocin–high-fat diet-induced MASH-fibrosis mice model (STAM mice) were prepared and characterized at SMC Laboratories, Inc. (Tokyo, Japan) as previously described (23). In brief, 200 µg of streptozotocin (STZ) were subcutaneously injected into C57BL/6J male mice at 2 days after birth. The mice were fed a low-fat ND (CE-2, CLEA Japan Inc.) until 4 wk of age and then fed an ad libitum high-fat diet (HFD: HFD32, CLEA Japan, Inc.) containing 32% total fat (60% kcal from fat) for up to 20 wk. STZ injection led to ∼60% of β cells being impaired when they were evaluated at 9 wk of age (3). For controls, age- and sex-matched C57BL/6J mice fed ad libitum ND or STZ-injected C57Bl/6J mice fed ad libitum ND were used. The frozen liver tissue was shipped to Central Virginia Veterans Affairs Healthcare System (Richmond VA Medical Center), and biomolecular analyses were performed.

#### Diet-induced MASH-fibrosis mice model.

Diet-induced MASH-fibrosis mice were prepared by Wang et al. (1). In brief, male C57BL/6J mice were fed a fructose-palmitate diet (Teklad, TD.160785) containing 1.25% cholesterol with sugar drinking water for 16 wk. Age- and sex-matched C57BL/6J mice fed ad libitum ND were the controls. The frozen liver tissue was sent to Central Virginia Veterans Affairs Healthcare System (Richmond VA Medical Center), and biomolecular analyses were performed.

### Human Subjects

Liver tissues from healthy individuals and patients with MASH were obtained from the National Institutes of Health (NIH)-sponsored Liver Tissue Cell Distribution System (LTCDS) at the University of Minnesota.

### Preparation of Primary Hepatocyte Culture and Infection with Ad-StarD5 (Gain-of-Function Study in Vitro)

Ad-CMV-hStarD5.MycDDK was prepared by Vector Biolabs as described in the *Materials* section. Primary hepatocytes were isolated from *StarD5*^−/−^ mice as previously described (24). They were plated on 6-well culture plates (500,000 cells/well) in William’s E media with insulin (0.25 U/mL) and dexamethasone (0.1 µM). The culture was incubated under 5% CO_2_ atmosphere at 37°C. The cells, 72 h after plating, were infected with the recombinant adenovirus encoding StarD5 (Ad-CMV-hStarD5.MycDDK) or Ad-eGFP control virus at multiplicity of infections (MOIs) of 500 and 2,500. Twenty-four hours after the infection, the cells were harvested for immunoblot and lipid analysis as described in the *Immunoblots* and *Lipid and Cholesterol Metabolite Quantifications* sections.

### Measurement of Serum Parameters

Serum glucose (Glu) levels were measured by the enzymatic procedures run on Siemens Vista 1500 instrumentation at the clinical laboratory of the Richmond VA Medical Center. Serum insulin was measured by the sensitive ELISA sandwich assay method using Crystal Chem Ultra-Sensitive Mouse Insulin ELISA kit (Elk Grove Village, IL) according to the manufacturer’s instructions. Homeostatic model assessment of insulin resistance (HOMA-IR) score as the determinant of insulin resistance was calculated as [glucose (mg/dL) × insulin (ng/mL) × 26/405].

### Lipid and Cholesterol Metabolite Quantifications

Hepatocytes (ca. 8 × 10^6^) harvested from the culture were resuspended in 0.8 mL of PBS and sonicated for 20 min in a glass tube. Then, proteinase K was added at 1 µg/mL final concentration and the cells were digested at 50°C for 5 h. A chloroform:methanol (2:1, vol/vol) solution of 16 mL was added and incubated at room temperature for 18 h. For livers, 100 mg of tissue was homogenized in lysate buffer (0.7 mL) containing 1% Nonidet P-40 (NP-40), 50 mM Tris-HCl pH 7.5, 150 mM NaCl, and 0.05% SDS. An aliquot of the homogenate (0.1 mL) was incubated with 3.5 mL of a chloroform:methanol (2:1, vol/vol) solution at room temperature for 18 h. After 1 mL of HPLC grade water was added, the solution was centrifuged at 3,000 rpm for 10 min. The chloroform (bottom) phase (containing the lipids) was collected and dried under nitrogen stream. The lipids were resuspended in isopropanol-10% Triton X-100 (1 mL), and an aliquot was subjected to the lipid measurements. Amplex Red Cholesterol Assay Kit and the Infinity Triglycerides Assay Kit (Thermo-Fisher Scientific, Cat. No. A12216 and 22421, respectively) were used for cholesterol and triglyceride measurement, respectively, according to the manufacturer’s instructions.

For quantification of liver 26-HC and 3βHCA, liver tissue (100 mg) was homogenized with cold methanol (1 mL) using Fischer brand Bead Mill 4 (Speed 5) for 120 s. An aliquot (20 µL) of the homogenate was mixed with the internal standard (IS) solution (60 µL), which consist of *d_5_*-25-HC (1 µg/mL), *d_6_*-26-HC (1 g/mL), and *d_5_*-3βHCA (1.0 µg/mL) in methanol. A 2-µL aliquot was injected to the Shimadzu LCMS-8060 CL liquid chromatography-mass spectrometry system as previously described (2).

### Hematoxylin and Eosin and Masson’s Trichrome Staining

For hematoxylin and eosin (H&E) and Masson’s trichrome staining, harvested liver tissue pieces were fixed in 4% neutral buffer formalin at room temperature and then embedded in paraffin, sectioned, and stained with hematoxylin and eosin or Masson’s trichrome at the Central Virginia Veterans Affairs Healthcare System (Richmond VA Medical Center) Pathology Laboratory. Images were taken in a Nikon Eclipse Ti inverted microscope using a ×40 objective (for H&E staining) or a ×20 objective (for Masson’s trichrome staining).

### Quantification of Masson’s Trichrome Staining

Quantification of Masson’s trichrome staining was performed using Image J software (https://imagej.net/software/fiji/) in liver slides from WT and *StarD5*^−/−^ mice. In each case, four fields from three different liver slides from WT and *StarD5*^−/−^ mice were used to determine the percent area positive for Masson’s trichrome staining.

### Histological Scoring System for Nonalcoholic Fatty Liver Disease

Nonalcoholic fatty liver disease (NAFLD) (now replaced by MASLD) activity score (NAS) was assessed following the guidelines described by Kleiner et al. (25) from the Nonalcoholic Steatohepatitis Clinical Research Network, which represents the sum of scores for steatosis (0–3), lobular inflammation (0–3), and ballooning (0–2), which in total ranges from 0 to 8. In general, NAS scores 0–2 are largely considered not diagnostic of MASH; scores 3–4 are evenly divided among cases considered not diagnostic, borderline, or positive for MASH; and scores of 5–8 occurs in cases largely considered diagnostic of MASH. Scores were determined by a blind reviewer.

### Sirius Red Staining

To visualize collagen deposition in liver samples from WT and *StarD5^−/−^* mice, formalin-fixed liver sections were stained using Picrosirius Red Stain Kit (Abcam, Cat. No. ab150681). In brief, sections were deparaffinized and hydrophilized with xylene, 100%–70% alcohol series, and RO water, and then treated with the Picrosirius red solution for 60 min. After two rinses with 0.5% acetic acid solution, the stained sections were dehydrated and cleared with two 100% alcohol series and then sealed with Permount Mounting Medium (Thermo Fisher Scientific, Cat. No. SP15-100) and used for observation. Bright-field images of Picrosirius red-stained sections were taken in a Nikon Eclipse Ti inverted microscope using a ×10 objective.

To visualize collagen deposition in liver samples from STAM mice, Bouin’s fixed liver sections were stained using Picrosirius red solution (FUJIFILM Wako Pure Chemical Corporation, Cat. No. 194-16202). In brief, sections were deparaffinized and hydrophilized with xylene, 100%–70% alcohol series, and RO water, and then treated with 0.03% Picrosirius red solution for 60 min. After being washed through 0.5% acetic acid solution and RO water, stained sections were dehydrated and cleared with 70%–100% alcohol series and xylene, then sealed with Entellan new (Merck, Germany) and used for observation. Bright-field images of Sirius red-stained sections were captured around the central vein using a digital camera (DFC295; Leica, Germany) with a ×20 objective.

### Hepatic VLDL Secretion Assay

To determine the hepatic VLDL secretion rate, *StarD5*^−/−^ mice and C57BL/6J mice were fasted for 4 h, followed by intraperitoneal injection of 100 µL of a 125-mg/mL Tyloxapol solution in sterilized PBS. Blood was collected from the cheek vein at time points of 0 (before Tyloxapol injection), 90, and 180 min. The plasma from the blood was obtained by centrifugation in Li-Heparin LH/1.3 tubes (Sarstedt) at 2,000 *g* for 10 min at room temperature. Triglycerides in plasma were measured using the Infinity Triglycerides assay kit as described in the *Lipid and Cholesterol Metabolite Quantifications* section.

### Nuclear and Cytoplasmic Extraction from Liver Samples

Nuclear and cytoplasmic fractions were extracted from livers (100 mg) of 8-wk-old male C57BL/6J and *StarD5*^−/−^ mice fed a ND for immunoblot analysis using the NE-PER Nuclear and Cytoplasmic Extraction Reagents kit following the manufacturer’s instructions. The protein concentrations of the fractions were quantified using the Protein Assay Dye Reagent (Bio-Rad).

### Immunoblots

Protein samples from cultured cells and tissues were separated on 12% SDS-PAGE gels and then transferred onto a PVDF membrane using a Bio-Rad semidry transfer cell apparatus. The membrane was transferred to a blocking solution, 5% nonfat dry milk in wash buffer (1.7 mM NaH_2_PO_4_, 8 mM Na_2_HPO_4_, 145 mM NaCl, 0.1% Tween 20) for 2 h at room temperature. The membrane was then incubated overnight in 2.5% nonfat dry milk in wash buffer containing a dilution of a primary antibody at 4°C, as indicated in Table 1. The membrane was then washed three times in wash buffer for 30 min at room temperature. After being washed, the membrane was incubated with a 1:2,000 dilution of the corresponding secondary antibody [horseradish peroxidase (HRP)-conjugated goat anti-rabbit IgG or goat anti-mouse IgG] in a 2.5% nonfat dry milk blocking solution in wash buffer for 1.5 h at room temperature. Finally, the membrane was washed three more times in wash buffer. Protein bands were visualized using SuperSignal West Pico Chemiluminescent Substrate and developed on a Bio-Rad ChemiDoc Touch imaging system.

To determine the hepatic apolipoprotein B (ApoB) levels by immunoblot, we made the following modifications to the general immunoblot protocol described earlier: *1*) the electrophoresis was performed in a 4%–20% gradient SDS-PAGE gel and *2*) the transfer of proteins was performed by wet transfer at 30 V for 16 h at 4°C, without methanol in the transfer buffer.

### Total RNA Extraction

Total RNA was extracted from livers (30 mg) of 8-wk-old male C57BL/6J and *StarD5*^−/−^ mice fed a ND [for RNA sequencing (RNA-Seq)] or from C57BL/6J and *StarD5*^−/−^ mice fed a Western diet for 18 wk (for fibrosis gene expression panel) and using the RNeasy Plus Mini Kit. The RNA concentration was quantified on a Nanodrop spectrophotometer.

### RNA-Seq and Bioinformatic Analysis

The RNA-Seq, with ribosomal RNA (rRNA) depletion and validation for purity and quantity, was performed by CD Genomics (Shirley, NY) using the Illumina HiSeq 50 platform. The raw data of the sequencing were filtered through a series of in-house filtration methods to obtain high-quality sequencing data for subsequent analysis. At the same time, Q30 and GC content of the clean data were calculated, and all the downstream analyses were based on the clean data with high quality. The filtered clean reads were mapped to reference genome by HISAT2 software. Gene expression data normalization and differential expression analysis were performed using the DESeq2. During the process, fold change ≥2 and false discovery rate (FDR) <0.05 were set as screening criteria. Fold change indicates the ratio of expression levels between two samples (groups), and false discovery rate is obtained by correcting the *P* value of the significant difference. Since the differential expression analysis of transcriptome sequencing is a test of independent statistical hypothesis for a large number of gene expression values, there is a problem of false positive. Therefore, the commonly used Benjamini–Hochberg calibration method was adopted for the differential expression analysis. The *P* value obtained from the test was corrected and finally FDR was used as a key indicator of differentially expressed genes. Genes with an adjusted *P* value ≤0.05 found by DESeq2 were assigned as differentially expressed. The heatmap was created using the online-based software Heatmapper (http://www.heatmapper.ca/expression/).

### Fibrosis Gene Expression Panel

Total RNA from livers was extracted and quantitated as described earlier, and a total of 200 ng of total RNA from each sample were analyzed using the commercial panel on 760 fibrosis-specific genes (nCounter Mouse Fibrosis V2 Panel) and the nCounter Analysis System (NanoString Technologies, Seattle, WA), respectively. Afterward, normalization of counts was performed using the nSolver analysis software version 3.0 (NanoString Technologies, Seattle, WA). Therefore, 10 internal reference genes were used, as predefined by the manufacturer. Well-established housekeeping genes, glucuronidase beta (*Gusb*) and phosphoglycerate kinase 1 (*Pgk1*), were designated as reference genes for standardization of measurements. Data were analyzed by ROSALIND (https://rosalind.bio/), with a HyperScale architecture developed by ROSALIND, Inc. (San Diego, CA). The heatmap was created using the online-based software Heatmapper (http://www.heatmapper.ca/expression/).

### Data Reproducibility, Quantification, and Statistical Analysis

All immunoblotting analyses were performed in Image Lab (Bio-Rad). Data are shown as means ± standard deviation (SD) and were analyzed using Prism 9 (GraphPad). Statistical significance of differences was determined by multiple unpaired, two-tailed Student’s *t* test with values of *P* ≤ 0.05 considered statistically significant.

## RESULTS

### StarD5 Deletion Triggers Lipid Accumulation in the Liver

**Figure 1.**
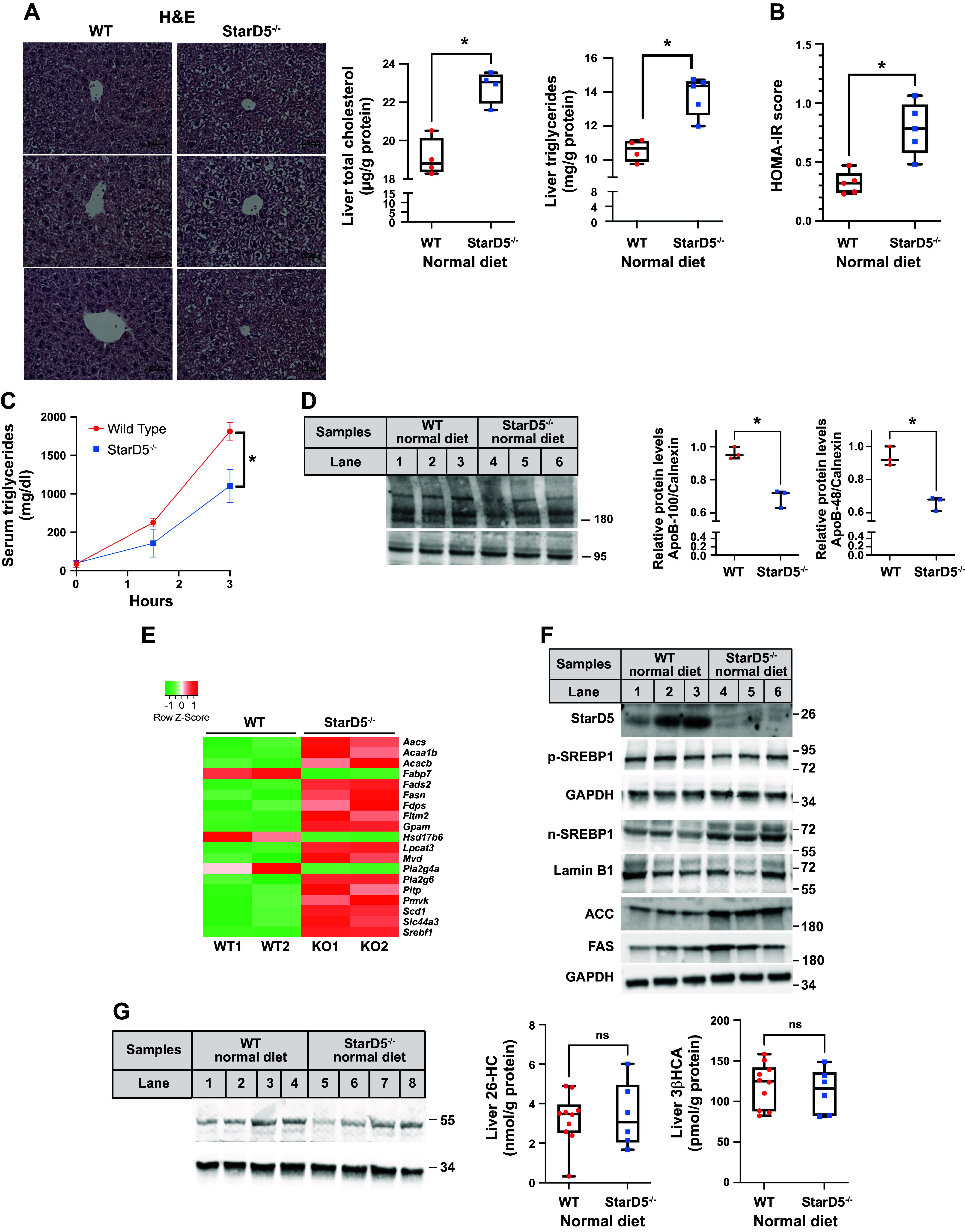
StarD5 deletion increases liver lipid content and leads to insulin resistance. *A*: sections from WT and *StarD5*^−/−^ mouse livers were stained with H&E and visualized with a ×40 objective lens. Representative images from three mice are shown. Note the extensive cytoplasmic vacuolization in the livers from *StarD5*^−/−^ animals. Total cholesterol and triglycerides were determined in livers of WT and *StarD5*^−/−^ mice fed a normal diet. *n* = 4 or 5. *B*: blood glucose and insulin values were used to calculate the homeostatic model assessment of insulin resistance (HOMA-IR) score. *StarD5*^−/−^ mice have higher HOMA-IR scores. *n* = 5. *C*: another group of mice (*n* = 5) were injected with Tyloxapol, then blood was collected at the indicated times after injection to quantify the TG levels. Wild-type mice have higher levels of triglycerides, indicating increased VLDL secretion into the blood than *StarD5*^−/−^ mice. *n* = 5. *D*: ApoB-100, ApoB-48, and calnexin (as loading control) were performed on liver homogenates from WT and *StarD5*^−/−^ mice, and relative expression levels were determined by densitometry of the ApoB-100 and ApoB-48 relative to calnexin. *E*: heatmap of differentially expressed genes in lipid metabolism in the livers of WT and *StarD5*^−/−^ mice. *F*: StarD5, precursor SREBP1 (p-SREBP1), nuclear SREBP1 (n-SREBP1), FAS, ACC, Lamin B1, and GAPDH (as loading controls) immunoblots were performed on liver homogenates from WT and *StarD5*^−/−^ mice. *G*: CYP7B1 and GAPDH (as loading control) immunoblots were performed on liver homogenates from WT and *StarD5*^−/−^ mice and liver (25 R)26-hydroxycholesterol (26-HC) and 3 b-hydroxy-5-choleste(25R)26-oic acid (3βHCA) levels in WT and *StarD5*^−/−^ mice. *n* = 6–10. ACC, acetyl CoA-carboxylase; ApoB-100, apolipoprotein B100; CYP7B1, oxysterol 7α-hydroxylase; FAS, fatty acids synthetase; H&E, hematoxylin and eosin; StarD5, steroidogenic acute regulatory-related lipid transfer domain 5; ns, not significant; TG, triglyceride; VLDL, very low-density lipoprotein; WT, wild type. *P < 0.05.

We have previously shown that the predominant effect of ablation of StarD5 in mice is the formation of intracellular vacuoles in hepatocytes (22) as revealed by H&E staining in livers that correspond to the accumulation of lipid droplets (Fig. 1*A*). Biochemical analysis of these livers revealed that the livers of *StarD5*^−/−^ mice had significantly increased levels of cholesterol (*P* ≤ 0.05) and triglycerides (*P* ≤ 0.05) compared with livers from WT mice (Fig. 1*A*) as previously demonstrated (22). In addition, the *StarD5*^−/−^ mice also had higher HOMA-IR scores as compared with WT mice (Fig. 1*B*), indicating developing IR. This difference in HOMA-IR was due to early increases in insulin levels in *StarD5*^−/−^ mice, as glucose levels in WT and *StarD5*^−/−^ mice were similar (Table 2), whereas insulin levels were elevated in the *StarD5*^−/−^ mice (1.129 ng/mL ± 0.4 compared with 0.468 ng/mL ± 0.17 in WT mice). The development of early insulin resistance did not alter other blood parameters of *StarD5*^−/−^, which were similar to those of WT mice fed a ND or WD (Table 2).

**Table 2.** Insulin development and levels in the steroidogenic acute regulatory-related lipid transfer domain 5 (StarD5^−/−^) mice compared with wild-type (WT) mice fed a normal diet or Western diet

Median, Min–Max	Normal Diet (*n* = 5)		18 Wk Western Diet (*n* = 5)	
	WT	*StarD5* ^−/−^	WT	*StarD5* ^−/−^
TG, mg/dL	69.3 (51–91)	58.1 (41–82)	78.5 (58–122)	72.5 (54–82)
Total cholesterol, mg/dL	112.5 (97–140)	94.1 (76–133)	306.2 (266–343)	246 (202–255)
HDL, mg/dL	98.8 (85–131)	87 (71–127)	272.2 (247–318)	236.5 (194–263)
ALT, IU/L	54.6 (30–78)	52.1 (25–90)	342.2 (277–436)	346 (227–435)
AST, IU/L	100.1 (45–182)	109.1 (45–141)	252.5 (210–303)	254 (131–330)
Glucose, IU/L	187.8 (87–245)	181.5 (140–265)	330.2 (294–368)	358.5 (329–399)

ALT, alanine aminotransferase; AST, aspartate transaminase; TG, triglyceride.

To determine what is triggering the accumulation of triglycerides in the livers of *StarD5*^−/−^ mice, we first quantitated the secretion of VLDL from the livers into the blood by monitoring triglyceride levels in plasma over time following the peritoneal injection of Tyloxapol, an effective inhibitor of VLDL disposal by inhibiting lipoprotein lipase activity. *StarD5*^−/−^ mice displayed significantly reduced triglyceride secretion rates compared with WT mice (Fig. 1*C*). Hepatic VLDL secretion rate is determined by several factors, including the availability of ApoB-100 (the major structural protein in VLDL) for the assembly of VLDL and the synthesis of triglycerides. Hepatic immunoblots revealed a significant decrease in immunodetectable levels of ApoB-100 and ApoB-48 in *StarD5*^−/−^ mice livers compared with WT mice livers (Fig. 1*D*). We also performed RNA-Seq analysis of livers from WT and *StarD5*^−/−^ mice to identify genes in the lipid metabolic pathways. The results revealed that the fatty acid synthesis pathway is particularly upregulated in *StarD5*^−/−^ mice as seen in the heatmap (Fig. 1*E*), suggesting the upregulation of *Srebf1*, the master transcriptional regulator of fatty acids biosynthesis, as well as some of the genes under its control including fatty acid synthetase (*Fas*) and acetyl CoA-carboxylase (*Acc*). Although total levels of SREBP-1 did not change much between the livers of WT and *StarD5*^−/−^ mice, nuclear levels of active SREBP1 (n-SREBP1) were increased in *StarD5*^−/−^ mice liver samples compared with WT mice liver samples (Fig. 1*F*). The elevated *Fas* and *Acc* gene levels were further confirmed by protein immunoblot (Fig. 1*F*).

**Figure 2.**
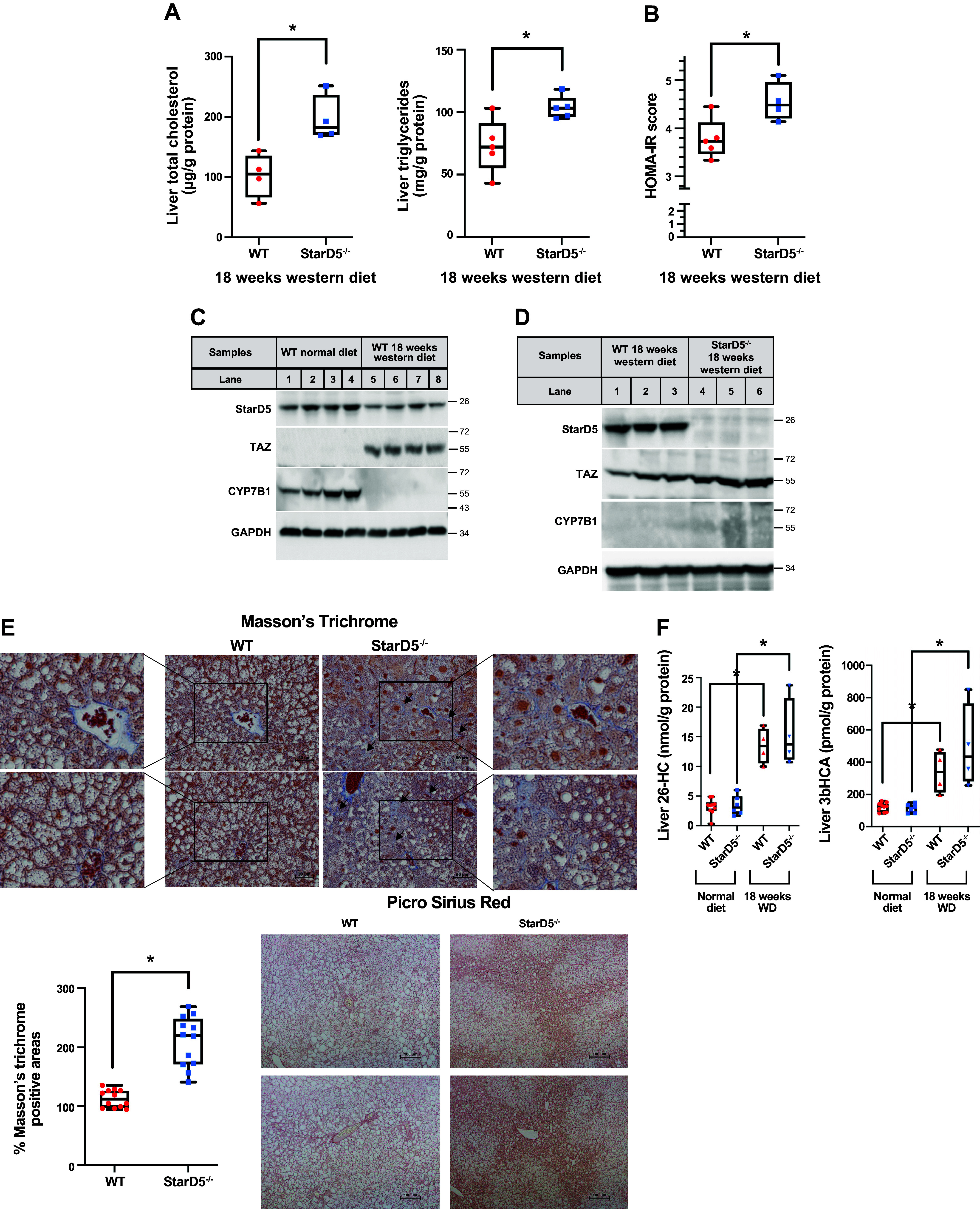
Western diet reduces expression of StarD5 in WT mice and increases TAZ expression in *StarD5*^−/−^ mice. *A*: total cholesterol and triglycerides were determined in the livers of WT and *StarD5*^−/−^ mice after 18 wk of Western diet (WD) feeding. *P < 0.01. *n* = 4 or 5. *B*: blood glucose and insulin values were used to calculate the homeostatic model assessment of insulin resistance (HOMA-IR) score of WT and *StarD5*^−/−^ mice after feeding a WD for 18 wk. *P < 0.02. *n* = 5. *C*: StarD5, Cyp7B1, and GAPDH (as loading control) immunoblots were performed on liver homogenates from WT mice fed regular chow or a Western diet for 18 wk. *D*: StarD5, TAZ, CYP7B1, and GAPDH (as loading control) immunoblots were performed on liver homogenates from WT and *StarD5*^−/−^ mice after being fed for 18 wk a Western diet. *E*: representative liver sections of WT and *StarD5*^−/−^ mice fed a Western diet for 18 wk were stained with Masson’s trichrome (arrows indicate areas of fibrosis) and Picrosirius red for collagen deposition/fibrosis; percentage area of positive staining for Masson’s trichrome. *P < 0.001. *n* = 12. *F*: liver 26-hydroxycholesterol (26-HC) and 3 b-hydroxy-5-cholestenoic acid (3βHCA) levels in WT and *StarD5*^−/−^ mice fed a normal diet and a Western diet for 18 wk. *n* = 4–8. *P < 0.01. CYP7B1, oxysterol 7α-hydroxylase; StarD5, steroidogenic acute regulatory-related lipid transfer domain 5; WT, wild type.

We additionally found unaltered expression of oxysterol 7α-hydroxylase (CYP7B1) in the *StarD5*^−/−^mice liver (Fig. 1*G*). *Cyp7b1*, which is responsive to IR, encodes an ER membrane protein catalyzing the hydroxylation of (*25 R*)26-hydroxycholesterol (26-HC) and 3β-hydroxy-5-cholesten-(25R)26-oic acid (3βHCA), major mitochondria-driven cholesterol metabolites. As expected, the livers of 18-wk-old *StarD5*^−/−^ mice did not show significant changes in levels of 26-HC or 3βHCA (Fig. 1*G*) as expression of CYP7B1 was found to be similar compared with WT mice. As shown in Fig. 2, the level of IR did not reach levels that correlated with lower CYP7B1 levels. It should be noted that without elevation of the mitochondrial cholesterol metabolites, no evidence of hepatotoxicity was noted. Thus, increased fatty acid synthesis, along with the reduced VLDL secretion, appears responsible for triglyceride accumulation in the livers of *StarD5*^−/−^ mice.

### Prolonged WD Feeding Decreases Expression of StarD5 in WT Mice

To study the roles of StarD5 in MASH pathogenesis, WT mice were challenged with ad libitum WD feeding for 18 wk. In response to the WD feeding, hepatic cholesterol levels (∼100 µg/g protein and ∼200 µg/g protein for WT and *StarD5*^−/−^ mice, respectively) were markedly elevated (Fig. 2*A*) from that of the ND-fed mice (Fig. 1*A*). Similarly, hepatic triglycerides (∼70 mg/g protein and ∼100 mg/g protein for WT and *StarD5*^−/−^ mice, respectively) were markedly elevated from that of the ND-fed mice. WD feeding also led to marked elevation of HOMA-IR score (Fig. 1*B* and Fig. 2*B*). Interestingly, immunoblotting found hepatic StarD5 protein was diminished in WT mice with the WD challenge (Fig. 2*C*). In addition, WD-fed WT mice had significantly lower hepatic CYP7B1 protein (Fig. 2*C*) coupled with elevated levels of hepatic 26-HC and 3βHCA (Fig. 2*D*). Moreover, TAZ, a transcriptional regulator that is increased in MASH livers and promotes fibrosis (1), was detected in the livers of WT mice fed WD, but not in those fed a ND (Fig. 2*C*).

### Prolonged WD Feeding Led to Increased Fibrosis in *StarD5^−/−^* Mice

Next, we challenged StarD5^−/−^ mice with *ad libitum* WD feeding for 18 wk. In response to the WD feeding, hepatic cholesterol and triglyceride levels were markedly elevated (Fig. 2*A*) from those of the ND-fed mice (Fig. 1*A*). These lipid accumulations were also more significant than those of WT mice livers. Similarly, WD feeding led to an elevated HOMA-IR score in *StarD5*^−/−^ mice (Fig. 2*B*), dramatically higher than that found in WD-fed WT mice, changes indicative of WD leading to an even greater increase in IR in *StarD5*^−/−^ mice. The immunoblots in Fig. 2*D* present hepatic StarD5, TAZ, and CYP7B1 protein levels of WD-fed *StarD5*^−/−^ and WD-fed WT mice. As expected, WD-fed *StarD5*^−/−^ mice did not express StarD5 in the liver. CYP7B1 was undetectable in both *StarD5*^−/−^ mice and WT WD-fed mice. TAZ levels were found to be higher in the livers of *StarD5*^−/−^ mice compared with livers from WT mice fed a WD, suggestive of more rapid onset and subsequent advanced fibrosis in the livers of *StarD5*^−/−^ mice. Masson’s trichrome and Sirius red staining of the liver sections showed a significant increase of collagen deposition in the livers of *StarD5*^−/−^ mice fed WD than those of WT counterparts (Fig. 2*E*). The observation correlates with higher amounts of TAZ in the *StarD5*^−/−^ mice liver. In addition, NAS scores were determined in the liver sections to assess MASH; while NAS scores for WT mice were 1–2, grades for *StarD5*^−/−^ mice liver slides were 4–5, indicating a more severe hepatic steatosis compared with WT mice.

**Figure 3.**
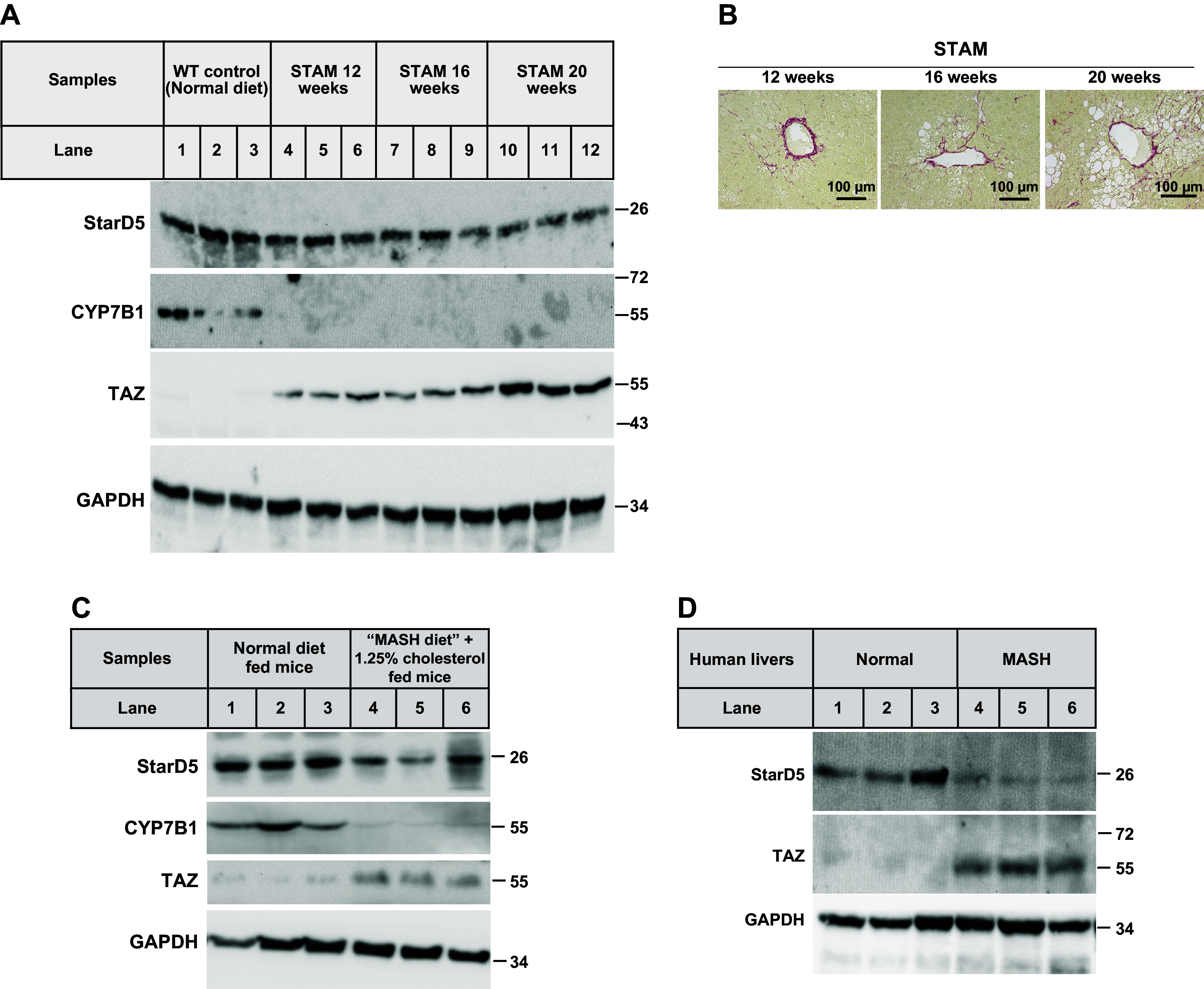
Development of MASH and fibrosis leads to the downregulation of StarD5 in two MASH-fibrosis mouse models and human MASH livers. *A*: StarD5, CYP7B1, TAZ, and GAPDH (as loading control) immunoblots were performed on liver homogenates from C57Bl/6 (WT) mice fed a normal diet or 12-, 16-, or 20-wk-old STAM mice. *B*: representative Sirius red staining of livers from mice injected with STZ and then fed a Western diet for 12, 16, or 20 wk. *C*: StarD5, CYP7B1, TAZ, and GAPDH (as loading control) immunoblots were performed on liver homogenates of WT mice fed a normal diet or a MASH diet + 1.25% cholesterol for 16 wk. *D*: StarD5, TAZ, and GAPDH (as loading control) immunoblots were performed on liver homogenates from normal and MASH human livers. CYP7B1, oxysterol 7α-hydroxylase; MASH, metabolic-associated steatohepatitis; STAM, streptozotocin–high-fat diet-induced MASH-fibrosis mice model; StarD5, steroidogenic acute regulatory-related lipid transfer domain 5; STZ, streptozotocin; WT, wild type.

Finally, levels of mitochondrial cholesterol metabolites (26-HC and 3βHCA) were measured in both WT and *StarD5*^−/−^ mice (Fig. 2*F*). Regardless of the genotype, WD feeding led to four- to fivefold elevation of hepatic 26-HC levels (15 nmol/g protein) from the corresponding ND-fed mice livers (3 nmol/g protein). Similarly, hepatic 3βHCA levels were three- to fivefold elevated (300–500 pmol/g protein) in the WD-fed mice livers from the corresponding ND-fed mice liver (100 pmol/g liver). Although the greatest effects would be expected with complete StarD5 knockout, it should be noted that WD induced greater than 50% suppression of StarD5, and coupled with the absence of detectable CYP7B1 in WT mice, led to similar increases in measured mitochondrial cholesterol metabolites, changes that more closely mimic a MASLD population (Fig. 3*D*).

### Streptozotocin-Induced Fibrosis (STAM), Diet-Induced MASH-Fibrosis Mice, and Livers of Patients with MASH Have Impaired StarD5 with Elevated TAZ in the Liver

Streptozotocin-induced fibrosis (STAM) is an increasingly studied MASLD mouse model (23). The STZ-treated mouse model was traditionally considered to be a cross between T1DM and T2DM and, therefore, not suitable for the study of MASLD as T2DM is classically associated with MASLD. However, recent studies have proven otherwise as intracellular insulin paucity (characteristic of both type 1 and 2) is what leads to low levels of CYP7B1 and the accumulation of toxic cholesterol metabolites, explaining the existence of MASLD progression with both types of diabetes. More specifically, the model appears to progress through similar stages of liver inflammation to fibrosis and cancer.

The mice reveal MASH-driven fibrosis at 9–10 wk of age that steadily progress to HCC over the following 10 wk. As MASH-fibrosis progresses, hepatic StarD5 was being decreased along with increased levels of TAZ (Fig. 3*A*). CYP7B1 was undetectable at all time points. Figure 3*B* presents the Sirius red staining of the liver sections at 12, 16, and 20 wk, with increased fibrosis over time and correlating with the levels of TAZ (Fig. 3*A*).

C57BL/6J mice fed with a fructose-palmitate diet containing 1.25% cholesterol for 16 wk were previously demonstrated to display significant hepatic fibrosis with elevated TAZ. We analyzed the liver tissues from the previous study (1) and were able to replicate the elevated expression of TAZ. These livers display reduced levels of StarD5 compared with normal livers (Fig. 3*C*). CYP7B1 was also markedly lower (Fig. 3*C*).

Livers from patients with MASH display decreased levels of StarD5 coupled with elevated TAZ compared with normal livers (*n* = 3) (Fig. 3*D*).

### Deletion of StarD5 Impacted Fibrosis-Relative Gene Expressions

**Figure 4.**
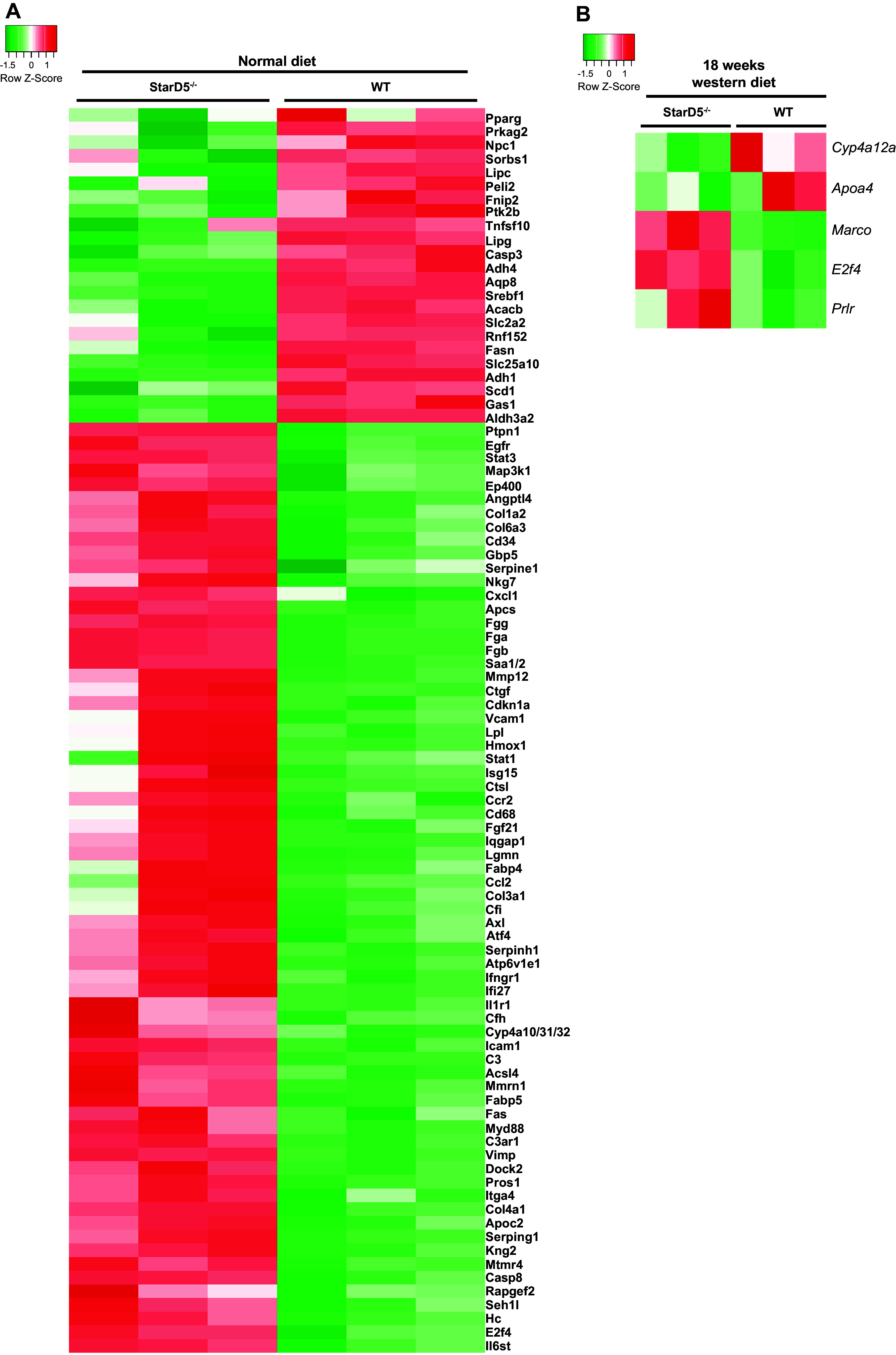
Deletion of StarD5 leads to a wide differential expression of genes in a fibrosis panel. *A*: heatmap of differentially expressed genes in a fibrosis panel in the livers of WT and *StarD5*^−/−^ mice. *n* = 3. *B*: heatmap of differentially expressed genes in a fibrosis panel in the livers of WT and *StarD5*^−/−^ mice after an 18-wk Western diet. *n* = 3. StarD5, steroidogenic acute regulatory-related lipid transfer domain 5; WT, wild type.

To further define the relevance of the lack of StarD5 in the development of fibrosis, we performed a fibrosis-relative gene expression panel using liver samples from *StarD5*^−/−^ and WT mice on ND and 18 wk of WD. When compared the livers from *StarD5*^−/−^ and WT mice fed an ND, a total of 91 genes changed in the fibrosis panel, with 23 upregulated genes and 68 downregulated genes in the livers of *StarD5*^−/−^ mice compared with WT mice livers (Fig. 4*A*). When the livers from WD-fed *StarD5*^−/−^ mice were compared with WD-fed WT mice, the number of differentially expressed genes narrowed down to only five genes (Fig. 4*B*).

### Ad-*StarD5* Overexpression Reduces Levels of Triglycerides in *StarD5*^−/−^ Mouse Hepatocytes

**Figure 5.**
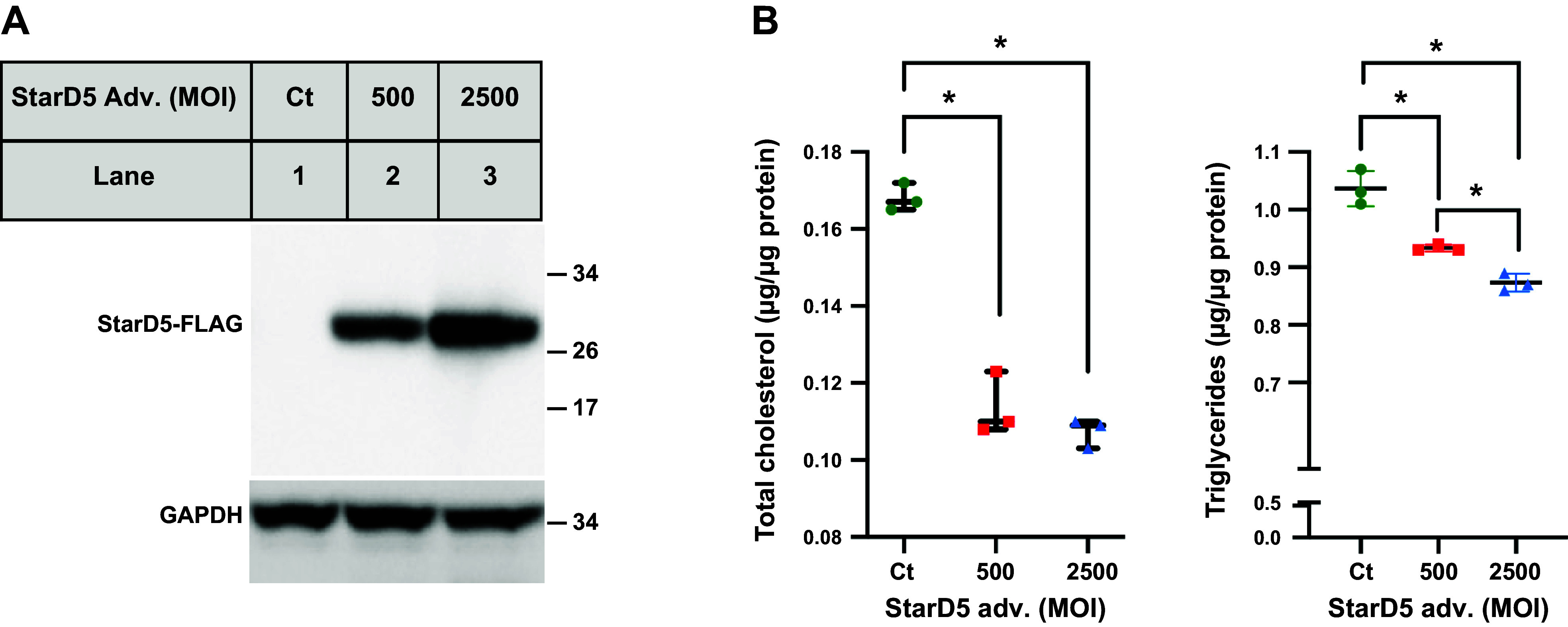
Restoring StarD5 expression in *StarD5*^−/−^ hepatocytes decreases TG levels. *A*: *StarD5*^−/−^ hepatocytes were infected with Ad-StarD5 or control adenovirus vector as indicated. Twenty-four hours after infection, hepatocytes were harvested, homogenates were prepared, and analyzed by immunoblot for StarD5-FLAG expression (GAPDH immunoblot was performed for loading control). *B*: total cholesterol and triglyceride levels were measured in the hepatocytes under the conditions described earlier. *n* = 3. *P < 0.005. StarD5, steroidogenic acute regulatory-related lipid transfer domain 5; TG, triglyceride.

To determine whether restoring the expression of StarD5 could reduce hepatic triglycerides, we studied adenovirus-mediated StarD5 overexpression in primary hepatocytes culture prepared from *StarD5*^−/−^ mice in a first approach. Primary hepatocyte culture was prepared as described in materials and methods. They were infected with an Ad-eGFP or an Ad-StarD5 at two different MOIs. The immunoblot shows that StarD5 protein levels were restored in a dose-dependent manner (Fig. 5*A*). As seen in Fig. 5*B*, levels of total cholesterol and triglycerides in hepatocytes from *StarD5*^−/−^ mice were reduced with restoring the expression of StarD5 (Fig. 5*B*).

### Restoring of Hepatic *StarD5* Improves Steatosis and IR in *StarD5*^−/−^ Mice

**Figure 6.**
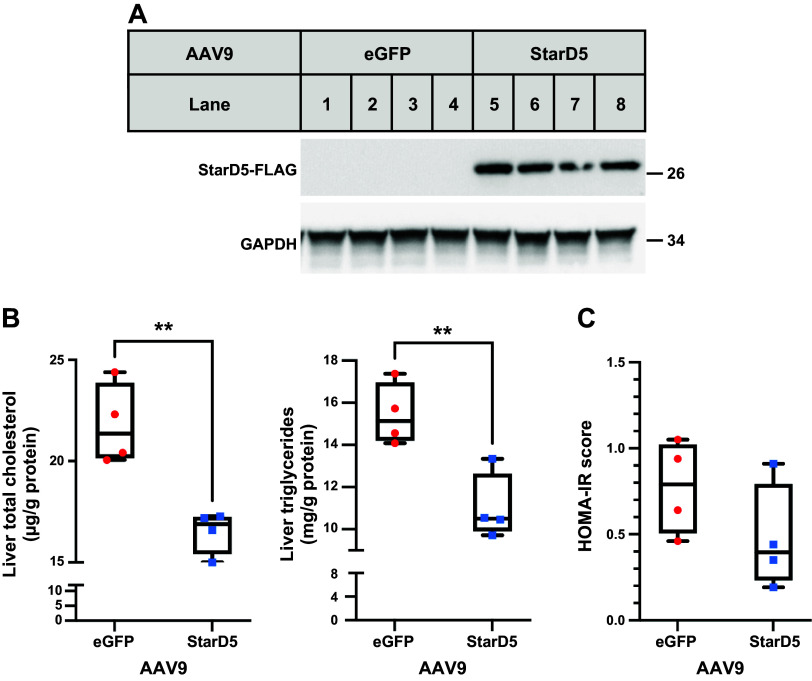
Injection of AAV9-StarD5 restores expression of StarD5 in the livers of *StarD5*^−/−^ mice and decreases TG levels and homeostatic model assessment of insulin resistance (HOMA-IR) score. *A*: mice were injected with AAV9-TGB-eGFP or AAV9-TBG-hStarD5.Myc.DDK (1 × 10^11^ MOI) as indicated. Three weeks later, livers were harvested, and homogenates were analyzed for StarD5 and GAPDH (loading control) expression. *B*: liver total cholesterol and triglyceride levels were determined in AAV9-TGB-hStarD5.Myc.DDK-injected *StarD5*^−/−^ mice. Liver total cholesterol and triglyceride levels were decreased compared with *StarD5*^−/−^ mice injected with AAV9-TGB-eGFP. *n* = 4. **P < 0.01. *C*: blood glucose and insulin values were used to calculate the homeostatic model assessment of insulin resistance (HOMA-IR) score in AAV9-TGB-hStarD5.Myc.DDK-injected *StarD5*^−/−^ mice. Restoring StarD5 expression in liver reduces HOMA-IR scores in *StarD5*^−/−^ mice. *n* = 4. eGFP, enhanced green fluorescent protein; MOI, multiplicity of infection; StarD5, steroidogenic acute regulatory-related lipid transfer domain 5.

To determine the effect of restoring the expression of StarD5 in vivo, AAV-mediated liver-selective StarD5 overexpression was studied in *StarD5*^−/−^ mice. Male *StarD5*^−/−^ mice were injected AAV9-TBG-hStarD5.MycDDK or the control counterpart AAV9-TBG-eGFP at 1 × 10^11^ genome copies/mouse. They were fed ad libitum ND for 3 wk. As seen in Fig. 6*A*, all mice that received the AAV9-TBG-hStarD5.MycDDK restore hepatic StarD5 to the same levels. Hepatic total cholesterol and triglyceride measurements (Fig. 6*B*) reveal that restoring the expression of StarD5 in the liver led to a significant decrease in total cholesterol and triglycerides. The calculated HOMA-IR score also trended down with restoring StarD5 (Fig. 6*C*), but the differences did not reach statistical significance due to the small sample size.

## DISCUSSION

Ablation of StarD5 led to increased accumulation of liver triglycerides secondary to hepatic upregulation in fatty acid synthesis (as an effect of increased SREBP1 activity) coupled with decreased rates of VLDL secretion (Fig. 1, *A*–*D*). Challenged with a WD, the *StarD5*^−/−^ mouse subsequently develops a more aggressive IR (Fig. 2*B*) and accelerated fibrosis (Fig. 2, *D* and *E*). Conversely, selectively restoring hepatocyte liver StarD5 expression led to a reduction in liver total cholesterol and triglycerides and to an improved IR (Fig. 6, *B* and *C*). These findings suggest that StarD5 plays a key physiological role in cholesterol and lipid housekeeping/homeostasis, with its downregulation leading to a proactive storage of lipids within the liver with nutritional abundance. However, its normal physiological downregulation with prolonged lipid excess promotes and accelerates liver fibrosis.

StarD5 has an important role in the transfer of cholesterol from the ER to the PM (10, 12, 26). This transfer of cholesterol is crucial for maintaining PM integrity and fluidity, as well as maintaining the correct levels of cholesterol in the ER (22, 27). Despite StarD5 being a cholesterol-transfer protein, we now show that depletion of StarD5 has profound consequences on lipid metabolism. More specifically, on triglycerides that accumulate in the liver of *StarD5*^−/−^ mice. Supportive evidence is provided by the upregulation of *Srebf-1* (the master regulator of fatty acid synthesis) (Fig. 1*E*) and of genes in the biosynthetic pathway of triglycerides, mainly *Fas* and *Acc* (Fig. 1, *E* and *F*). Unclear is what leads to upregulation of *Srebf1*. Some indication could come from differences on the levels of oxysterols, known to regulate *Srebf1* through liver X receptor (LXR) (28), but this is not supported in this case as levels of CYP7B1 protein and oxysterols (Fig. 1*G*) remain similar in the livers of *StarD5*^−/−^ mice compared with WT mice, therefore having no effect on the regulation of Srebf-1, as we have shown when different mitochondrial cholesterol metabolites are increased (29). Another possibility for the upregulation of Srebf-1 in *StarD5*^−/−^ mice is the increased levels of insulin, which leads to the transport of SREBP-1 from the ER to the Golgi, where it is processed by proteases, and finally to the nucleus to induce the expression of lipogenic genes (30). The other factor that clearly plays a role in the triglyceride accumulation in the liver of *StarD5*^−/−^ mice is their reduced rate of VLDL secretion from the liver (Fig. 1*C*), which reduces triglycerides being shipped out of the liver and being stored instead. What remains unknown is the element that leads to the decrease of VLDL secretion, as hepatic VLDL secretion rate Is determined by multiple factors such as free fatty acid influx to the liver, rate of hepatic triglycerides biosynthesis, and VLDL packaging (31).

Reduced ApoB levels in the livers of *StarD5*^−/−^ mice (Fig. 1*D*), coupled with increased triglyceride synthesis (Fig. 1, *E* and *F*), appear to be able to drive steatosis in *StarD5*^−/−^ mouse livers. Still, how the absence of StarD5 leads to the reduction of ApoB-100 and ApoB-48 is unknown. The current model for the lipidation of VLDL proposes two steps (32), with the first step taking place in the rough ER, from where ApoB is translocated to the ER lumen (33–35). If this first step is interrupted, as it happens under conditions of ApoB or microsomal triglyceride transfer protein (MTTP) deficiency, the integrity of the nascent particle is compromised and results in its degradation (32, 36, 37). We have shown that the absence of StarD5 leads to changes in cholesterol levels and subsequently in the fluidity of the PM and the ER membranes (22). These changes in the lipid composition of the ER membrane alters not only the physical behavior of the membrane but also how lipids interact with membrane proteins (38). In this case, it likely leads to increased degradation and decreased lipidation of ApoB/VLDL (34, 35).

It should be noted that continued aggressive overfeeding overwhelmed the livers of both WT and *StarD5*^−/−^ mice. Furthermore, WD feeding in and of itself led to a marked downregulation in StarD5 levels of expression, somewhat dampening comparative changes with *StarD5*^−/−^ mice. The faster development of MASH and/or fibrosis in the livers of *StarD5*^−/−^ mice was coupled with complementary changes in gene expression observed in the fibrosis panel (Fig. 4), comparing WT and *StarD5*^−/−^ mice on a normal diet. With regard to fibrosis, following WD feeding, all livers showed expression of TAZ, but at higher levels in the livers from *StarD5*^−/−^ mice (Fig. 2*D*). Figure 2*E* shows the histological findings of increased fibrosis coupled with the increase in TAZ levels. Similar trends correlating increased histologic fibrosis to TAZ levels were also found in the STAM model (Fig. 3*A*), findings supported by human MASH samples (Fig. 3*D*). Importantly, correlative increases in oxysterol levels paralleled the increase in TAZ and fibrosis.

Recently, we showed that development of IR with WD feeding in several animal models was temporally associated with suppression of insulin-responsive *Cyp7b1*, a subsequent increase in oxysterol levels, and initiation of liver toxicity, a progression previously observed in humans lacking *Cyp7b1* (2, 3, 39). The findings of this paper are supportive of those findings, as TAZ upregulation was closely tied to CYP7B1 suppression and increased oxysterol levels. Although measurement of TAZ has never been analyzed in humans lacking *Cyp7b1*, it is reasonable to hypothesize that activation of TAZ can be directly driven by oxysterols as well.

It should be noted that the metabolites described as the leading candidates for initiating and perpetuating the drive of MASLD to MASH *1*) present as toxic entities that initiate and lead to a multitude of pathophysiological responses; *2*) are not toxic metabolic products at physiological levels; and *3*) because of their membrane-disruptive properties, true pathological effects cannot be discerned from membrane-disruptive effects by adding *in vitro* or *in vivo* models. Therefore, the rationale in this study for coupling early *in vivo* responses to their increased endogenous synthesis to more chronic ones.

In the presence of MASH, StarD5’s reduced expression (Fig. 2*C* and Fig. 3, *A*, *C*, and *D*) might also decrease the transport of cholesterol to the PM, leading to its accumulation in the ER while potentiating triglyceride accumulation and exacerbation of IR. This might serve as a countermeasure to the PM to ER transport of cholesterol by ASTER-B/C (1, 40), furthering an environment leading to unfolded protein response (UPR) associated with more severe MASH (41).

In conclusion, provided is the evidence of StarD5’s role in the control of both cholesterol and lipid homeostasis within the liver. Downregulation of StarD5 that occurs with lipid abundance triggers what might be initially considered a normal physiological response to store triglycerides for future energy needs. However, continued suppression of StarD5 with a continuing supply of lipid excess promotes continued lipid storage, development of IR, likely toxic levels of cholesterol metabolite accumulation, and accelerated fibrosis, all hallmarks of lipid-related disease of the liver (MASLD/MASH). These findings support the role of cholesterol metabolites as initiators of toxicity followed by sequential liver pathology. Therefore, being able to maintain normal expression levels of StarD5 may represent a key element in the prevention of fatty liver disease.

## DATA AVAILABILITY

Data availability and requests for resources and reagents should be directed to and will be fulfilled by the corresponding author D. Rodriguez-Agudo: daniel.rodriguezagudo@vcuhealth.org.

## GRANTS

This work was supported by United States Department of Veterans Affairs: Veterans Administration Merit Review Award I01 BX005895-01A2 (to W.M.P. and D.R.-A.), Virginia Commonwealth University VETAR Award 411456 (to D. R.-A.), and the Virginia Commonwealth University Department of Internal Medicine Pilot Project Fund 411864 (to G.K.).

## DISCLOSURES

No conflicts of interest, financial or otherwise, are declared by the authors.

## AUTHOR CONTRIBUTIONS

G.K., N.B.-K., T.H., W.M.P., and D.R.-A. conceived and designed research; G.K., K.M., N.B.-K., T.H., W.M.P., and D.R.-A. performed experiments; G.K., K.M., N.B.-K., T.H., W.M.P., and D.R.-A. analyzed data; G.K., K.M., N.B.-K., T.H., W.M.P., and D.R.-A. interpreted results of experiments; G.K. and D.R.-A. prepared figures; G.K., N.B.-K., T.H., W.M.P., and D.R.-A. drafted manuscript; G.K., K.M., N.B.-K., T.H., W.M.P., and D.R.-A. edited and revised manuscript; G.K., K.M., N.B.-K., T.H., W.M.P., and D.R.-A. approved final version of manuscript.
